# Spread of Adenovirus to Geographically Dispersed Military Installations, May–October 2007

**DOI:** 10.3201/eid1605.091633

**Published:** 2010-05

**Authors:** Jill S. Trei, Natalie M. Johns, Jason L. Garner, Lawrence B. Noel, Brian V. Ortman, Kari L. Ensz, Matthew C. Johns, Michel L. Bunning, Joel C. Gaydos

**Affiliations:** United States Air Force School of Aerospace Medicine, San Antonio, Texas, USA (J.S. Trei, N.M. Johns, J.L. Garner, M.C. Johns); Air Education and Training Command, San Antonio (L.B. Noel, B.V. Ortman); Sheppard Air Force Base, Wichita Falls, Texas, USA (K.L. Ensz); Lackland Air Force Base, San Antonio (M.L. Bunning); Armed Forces Health Surveillance Center, Silver Spring, Maryland, USA (J.C. Gaydos); 1 Current affiliation: Allina Hospitals & Clinics, Minneapolis, MN, USA.

**Keywords:** Viruses, adenovirus, surveillance, respiratory disease, epidemiology, research

## Abstract

In mid-May 2007, a respiratory disease outbreak associated with adenovirus, serotype B14 (Ad14), was recognized at a large military basic training facility in Texas. The affected population was highly mobile; after the 6-week basic training course, trainees immediately dispersed to advanced training sites worldwide. Accordingly, enhanced surveillance and control efforts were instituted at sites receiving the most trainees. Specimens from patients with pneumonia or febrile respiratory illness were tested for respiratory pathogens by using cultures and reverse transcription–PCR. During May through October 2007, a total of 959 specimens were collected from 21 sites; 43.1% were adenovirus positive; the Ad14 serotype accounted for 95.3% of adenovirus isolates. Ad14 was identified at 8 sites in California, Florida, Mississippi, Texas, and South Korea. Ad14 spread readily to secondary sites after the initial outbreak. Military and civilian planners must consider how best to control the spread of infectious respiratory diseases in highly mobile populations traveling between diverse geographic locations.

## Abstract 2

Adenovirus serotype B14 spread readily to other sites after an initial outbreak at a military basic training facility.

Adenovirus (Ad)–associated acute respiratory disease (AdARD) epidemics have been widely reported among recruits at US Department of Defense (DoD) training centers (*1*–*5*). Vaccines targeting Ad4 and Ad7, the most common serotypes associated with these illnesses, were used among United States military trainees from 1971 through early 1999, when the supply was exhausted following cessation of vaccine production in 1996 (*1*). Because of the historically high negative effects of respiratory disease and the discontinuation of vaccine, the DoD initiated a population-based, active surveillance program in 1996 to track acute respiratory disease (ARD) activity among recruits at 8 military training centers, including the Air Force’s only recruit training center at Lackland Air Force Base (AFB) in San Antonio, Texas (*1*,*6*,*7*).

Lackland AFB admits 400–800 new basic military trainees (BMTs) per week; ≈35,000 BMTs graduate annually. BMTs are assigned to flights of 45–65 persons during the 6.5-week training program. All flight members train, eat, and sleep as a unit and are housed in 1 large open-bay facility. According to DoD surveillance data, during January 2005–January 2007 Lackland AFB experienced relatively mild ARD activity among BMTs; rates ranged from 0.1–0.7 cases per 100 recruit-weeks (US Naval Health Research Center, unpub. data). No adenovirus-positive specimens from Lackland AFB were serotyped during 2005, and only 4 were serotyped during 2006; serotypes included 1 Ad21, 1 AdC, and 1 Ad3. One specimen showed an Ad14/Ad21 co-infection (*8*). Adenovirus serotype B14 (Ad14) was detected at Lackland AFB for the first time in 2006; in that same year, Ad14 was also detected at 3 other DoD training centers (*8*).

Beginning in February 2007, an outbreak of respiratory illness associated with Ad14 occurred among Lackland AFB BMTs. During the height of the outbreak in June 2007, ARD rates exceeded 2.0 cases per 100 recruit-weeks (Naval Health Research Center, unpub. data). Most cases involved only mild, acute, febrile, respiratory illness. However, during April–October 2007, 27 patients were hospitalized with pneumonia and more severe sequelae; some patients required intensive care. All these patients were found to be adenovirus positive, and 20 (74.1%) had positive tests for the Ad14 subtype. The recognition of these more severe cases prompted an investigation and enhanced surveillance to describe the clinical and epidemiologic characteristics of Ad14 in this population. Laboratory results from early in the investigation indicated that 63% of ARD-related respiratory specimens collected from BMTs were positive for adenovirus and that 90% of adenovirus infections were the Ad14 subtype (*9*). Most BMTs became ill with adenovirus in training weeks 4 and 5 (US Naval Health Research Center, unpub. data) and may have still been infectious after graduation because virus shedding can occur in respiratory secretions and feces for several weeks (*10*–*12*).

We modeled the transmission of Ad14 through 2 hypothetical flights containing 50 BMTs each (Figure 1) by using data based on actual laboratory results and epidemiologic findings from Lackland AFB; our model indicated that >50% of BMTs, during the height of the outbreak, were infected with Ad14 over the course of the 6.5 week training period ( *9;* Naval Health Research Center, unpub. data). At the end of basic training, with the conservative assumption that recovering patients shed virus for up to 1 month, ≈28% of BMTs were still infectious at graduation and in the following days or weeks. Given the likelihood that some BMTs were still ill or shedding Ad14 after completing basic training, response and control efforts had to account for the high mobility of this population.

**Figure 1:**
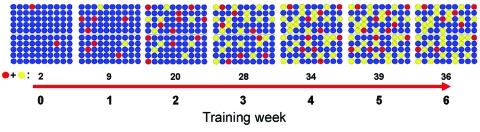
Evolving adenovirus subtype B14 incidence rate per 100 US Air Force basic military trainees over 6.5 weeks of basic training, based on epidemiologic and laboratory surveillance data. Red circles, acutely ill; yellow circles, recovering/possibly infectious; blue circles, well.

Following graduation, students immediately dispersed to >130 secondary DoD sites for advanced training (Figure 2); most went to a few large Air Force training centers in the United States, while a few went to smaller sites worldwide. Secondary training sites, including Sheppard AFB (Wichita Falls, TX, USA), Goodfellow AFB (San Angelo, TX, USA), and Keesler AFB (Biloxi, MS, USA), began reporting increased ARD among their trainees in mid to late May 2007. We report the spread of Ad14 to secondary training installations and subsequent response efforts, following the Lackland AFB outbreak, from May 25 through October 31, 2007.

**Figure 2:**
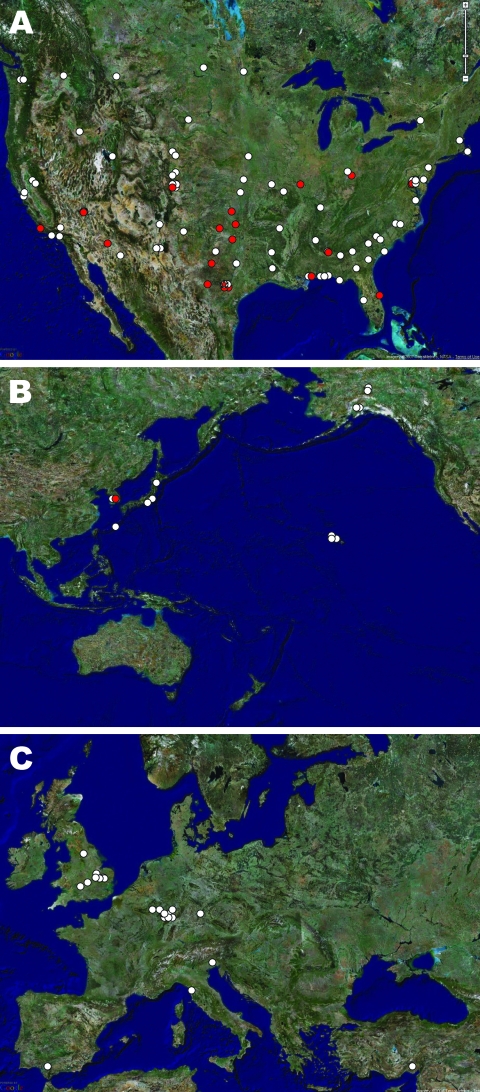
Locations of military sites that received US Air Force basic military training graduates for secondary training in North America (A), the Pacific region (B), and Europe and the Middle East (C). Red indicates locations that submitted specimens as part of adenovirus surveillance. Star in panel A indicates Lackland Air Force Base, Texas, USA. Maps generated by using TerraMetrics (www.terrametrics.com).

## Methods

### Surveillance

In late May 2007, enhanced, active ARD surveillance was initiated at 12 military installations that received basic training graduates, including 5 Air Force secondary training sites that received the most graduates from Lackland AFB: Sheppard AFB, (28.4% of BMT graduates); Keesler AFB, (16.4%); Goodfellow AFB, (3.8%); Hurlburt Field, Florida (1.8%); and Brooks City-Base, Texas (0.7%). In total, the 12 sites participating in enhanced surveillance efforts received 54.2% of BMT graduates moving to non–Lackland AFB sites for their secondary training.

Staff from the US Air Force School of Aerospace Medicine (USAFSAM) sent respiratory specimen collection kits and educational materials to the secondary sites that had cases, created a website to disseminate information, and encouraged participation through regular email correspondence. Investigators at USAFSAM enhanced surveillance efforts by directing efforts to sites with suspected cases but decreasing or few specimen submissions.

Nasal wash, nasopharyngeal swab, and oropharyngeal (OP) swab specimens were collected from patients meeting the ARD case definition May 25–October 31, 2007, and sent to USAFSAM in viral Universal Transport Medium (Copan, Brescia, Italy). The ARD case definition included fever of >100.5°F with a cough or sore throat or evidence of pneumonia. Routine patient surveys that accompanied laboratory specimens were reviewed to obtain patients’ demographic data, signs and symptoms, additional clinical information, travel history, and lost training days.

Additionally, staff from USAFSAM and the Air Education and Training Command headquarters coordinated the public health response and provided guidance on prevention, enhanced surveillance, and control efforts. Sites not included in initial enhanced surveillance efforts were also invited to send specimens collected from patients whose conditions met the case definition. By using laboratory surveillance data, weekly ARD and AdARD trends were tracked at the 3 main sites reporting increased ARD, Sheppard AFB, Keesler AFB, and Goodfellow AFB.

### Laboratory Methods

Specimens were tested by traditional viral culture, shell vial culture (R-Mix; Diagnostic Hybrids, Athens, OH, USA), and, beginning July 25, 2007, reverse transcription–PCR (RT-PCR) for subtype B14. Most adenovirus culture–positive specimens submitted between May 26 and July 25, 2007 were tested for Ad14 after the test capability became available. Viral and shell vial culture identified adenovirus, influenza, parainfluenza viruses 1–3, respiratory syncytial virus, and rhinovirus, as well as herpes simplex virus 1 and enterovirus after additional evaluation. Tube cultures were examined for 10 days for cytopathologic effects, and cells from the shell vial cultures were stained with pooled fluorescent antibodies and virus-specific monoclonal antibodies. The procedure and hexon-specific oligonucleotides for the adenovirus B14-specific monoplex RT-PCR were adapted from a US Naval Health Research Center protocol (*8*,*13*). On nasal wash and OP swab specimens and adenovirus isolates, DNA was extracted from the transport media and amplified by RT-PCR. The resulting RT-PCR products were then purified by using Millipore (Billerica, MA, USA) microcolumns and subsequently analyzed by agarose gel electrophoresis, DNA sequencing, or both. The analyte-specific reagent (ASR) primers and laboratory-developed diagnostic assay were used in accordance with requirements specified by the College of American Pathologists for use as part of molecular diagnostics testing performed at USAFSAM.

### Prevention and Control

In addition to adopting prevention and control measures to mitigate transmission within its own training population (*9*), Lackland AFB officials initiated actions to reduce spread to secondary training sites. Personnel screened outgoing BMTs from Lackland AFB for fever by placing chemical temperature dots on the forehead. Students with a temperature >100.5°F were held back from travel and housed in a medical-hold dormitory until their measured temperatures dropped below 100.5°F for 24 hours. Secondary training sites also adopted prevention and control measures to help incoming students and other assigned active duty members remain as healthy as possible.

Because it typically receives the most BMT graduates, Sheppard AFB instituted more aggressive case-finding procedures and prevention measures than any other secondary training site and fully implemented these actions by June 8, 2007, 12 days after enhanced surveillance efforts began. Their prevention efforts are described here; several other secondary sites instituted similar practices. All students arriving from Lackland AFB were screened for a measured fever >100.5°F and administered a questionnaire during in-processing. Students suspected of having ARD were further screened by a healthcare provider and sent to the clinic for treatment and testing as appropriate. Students with ARD were issued masks, grouped with other ARD students, placed on quarters (confined to their living area and restricted from participating in all work and leisure activities), and removed from all training activities. Students on quarters were not allowed to enter dining halls, and meals were instead brought to their rooms. Students were reevaluated by a healthcare provider after 24 hours on quarters and returned to duty if afebrile.

Sheppard AFB mandated that a virucidal cleaning agent be used several times per day to sanitize high-touch surfaces in facilities, including dining halls, classrooms, dormitories, buses, taxis, the post office, and other student-frequented establishments. In addition, hand washing and use of hand sanitizer were highly encouraged and closely monitored.

Upon completion of the training program, outgoing students were also screened for ARD by using the same questionnaire and a documented temperature. All students suspected of having ARD were placed on medical hold and evaluated by a physician to determine whether treatment was needed. After 24 hours they were reevaluated and released to travel if afebrile.

## Results

From May 25 through October 31, 2007, USAFSAM received 959 respiratory specimens from the 12 secondary training sites that initially participated in enhanced surveillance and from 9 additional sites (Table 1). Adenovirus accounted for 413 (89.8%) of the 460 specimens with known etiologic agents; the other viruses identified included parainfluenza (31 [6.7%]), influenza type A (5 [1.1%]), respiratory syncytial virus (2 [0.4%]), and enterovirus (1 [0.2%]). Among the specimens that were culture positive for adenovirus, 358 (86.7%) were tested for Ad14, of which 341 (95.3%) were positive. Ad14 was identified at 8 secondary sites located in California, Florida, Mississippi, Texas, and South Korea; collection dates of the first Ad14-positive specimen at each site ranged from May 30 through October 30. Most patients (331 [97.1%]) with confirmed Ad14 infection were advanced training students, while 9 (2.6%) infections occurred in active duty members outside the training population, and 1 (0.03%) occurred in a dependent child.

**Table 1:** Summary of results from respiratory specimens received from USAF secondary training bases, May 25–October 31, 2007*

Site†	No. specimens	Adenovirus not otherwise specified, no. (%)	Ad14, no. (%)	Date first Ad14-positive specimen collected
Altus AFB, OK	2	0	0	–
Andrews AFB, MD	12	1 (8)	0	–
Bolling AFB, DC	1	0	0	–
Brooks City-Base, TX	10	1 (10)	1 (100)	Jun 30
Goodfellow AFB, TX	71	37 (52)	19 (51)	Jun 1
Hurlburt Field, FL	3	2 (67)	2 (100)	Oct 22
Keesler AFB, MS	85	46 (54)	38 (83)	May 31
Laughlin AFB, TX	11	2 (18)	0	–
Luke AFB, AZ	3	1 (33)	0	–
Maxwell AFB, AL	23	1 (4)	0	–
Nellis AFB, NV	3	0	0	–
Osan AB, South Korea	6	1 (17)	1 (100)	Jun 19
Patrick AFB, FL	2	0	0	–
Randolph AFB, TX	9	1 (11)	1 (100)	Oct 30
Scott AFB, IL	9	2 (22)	0	–
Sheppard AFB, TX	683	309 (45)	273 (88)	May 30
Tinker AFB, OK	6	2 (33)	0	–
USAF Academy, CO	6	0	0	–
Vance AFB, OK	0	0	0	–
Vandenberg AFB, CA	10	7 (70)	6 (86)	Jun 14
Wright-Patterson AFB, OH	4	0	0	–
Total	959	413 (43)	341 (83)	

*USAF, United States Air Force; AD14, adenovirus B14; AFB, Air Force Base; OK, Oklahoma; MD, Maryland; DC, District of Columbia; TX, Texas; FL, Florida; MS, Mississippi; AZ, Arizona; AL, Alabama; NV, Nevada; AB, Air Base; IL, Illinois; CO, Colorado; CA, California; OH, Ohio. †All sites located in the United States except Osan AB (South Korea).

Patient survey data were available for 538 of the 959 (56.1%) patients from whom specimens were collected; of these, 220 (40.9%) were Ad14 positive. The following results are only for those 220 patients with confirmed Ad14 infection and available patient survey data (Table 2). Patient ages spanned 17–29 years, though most (183 [84.7%]) patients were 18–22 years of age; the median age was 19 years. In addition, most (197 [89.5%]) patients were male. Regarding patient symptoms, the median temperature recorded was 101.0°F. The most common signs and symptoms reported by patients were sore throat (90.9%), chills (83.2%), fatigue (78.6%), cough (78.2%), headache (75.9%), body aches (70.0%), and nasal congestion (61.4%). One patient was hospitalized with pneumonia and recovered fully without complications. A total of 191 (86.8%) patients were placed on quarters. Of the 125 patients for whom length of quarters information was available, most (108 [86.4%]) were placed on quarters for 24 hours. In addition, 147 (66.8%) patients had recently traveled; of these, most (143 [97.3%]) had recently traveled from Lackland AFB.

**Table 2:** Demographic data, symptoms, and other information collected from 220 patients with positive test results for adenovirus serotype B14, May 25–October 31, 2007*

Parameter	Value
Median age, y (range), n = 216	19 (17–29)
Gender	
F	23 (10.5)
M	197 (89.5)
Base where stationed	
Goodfellow AFB, TX	8 (3.6)
Hurlburt Field, FL	2 (0.9)
Keesler AFB, MS	16 (7.3)
Sheppard AFB, TX	188 (85.5)
Vandenberg AFB, CA	6 (2.7)
Signs and symptoms	
Body aches	154 (70.0)
Chest pain	38 (17.3)
Chills	183 (83.2)
Conjunctivitis	24 (10.9)
Cough	172 (78.2)
Diarrhea	42 (19.1)
Dyspnea	36 (16.4)
Earache	60 (27.3)
Fatigue	173 (78.6)
Headache	167 (75.9)
Runny nose	93 (42.3)
Sinus congestion	135 (61.4)
Sore throat	200 (90.9)
Stiffness	89 (40.5)
Vomiting	39 (17.7)
Median clinical temperature	101°F
Placed on quarters	191 (86.8)
Time on quarters, n = 125	
24 h	108 (86.4)
48 h	13 (10.4)
72 h	4 (3.2)
Hospitalized	1 (0.5)
Received influenza vaccine	68 (30.9)
Recent travel	147 (66.8)
Recent travel locations, n = 147	
Lackland AFB, TX	143 (97.3)
Keesler AFB, MS	2 (1.3)
Albuquerque, NM	1 (0.7)
Panama City, FL	1 (0.7)

*Values are no. (%) except as specified. Complete information not available for all patients; n values given when below 220. All locations in United States. AFB, Air Force Base; TX, Texas; FL, Florida; MS, Mississippi; CA, California; NM, New Mexico.

**Figure 3:**
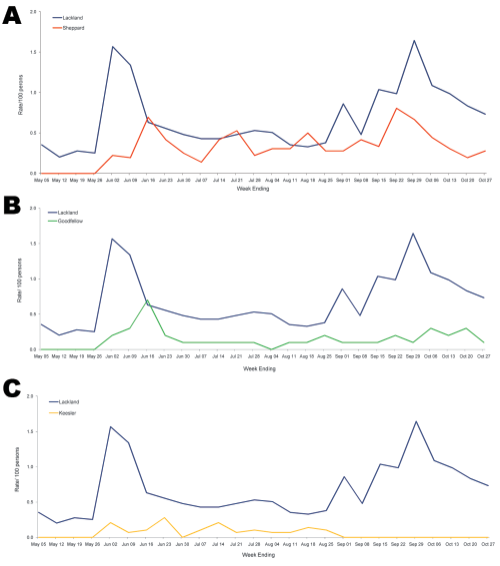
Rates of confirmed adenovirus for secondary training students at Sheppard Air Force Base, Texas, USA (A); Goodfellow Air Force Base, Texas, USA (B); and Keesler Air Force Base, Mississippi, USA (C), compared with rates for basic military trainees at Lackland Air Force Base, Texas, USA, May 25–October 31, 2007.

At the 3 secondary sites receiving the most BMT graduates, Sheppard AFB, Goodfellow AFB, and Keesler AFB, AdARD incidence rates among active duty personnel were tracked and compared with concurrent rates calculated at Lackland AFB (Figure 3). AdARD rates at Lackland AFB ranged from 0.1–2.0 cases per 100 personnel, with 2 peaks in June and September 2007. AdARD activity at Sheppard AFB waxed and waned throughout the surveillance period, ranging from 0.2–0.8 ARD cases per 100 personnel. The largest peak of activity occurred on September 22, 2007, 2 weeks following the onset of Lackland AFB’s second wave of activity. However, this AdARD activity was short lived, decreasing over a course of 4 weeks to 0.2 cases per 100 personnel. Activity at Goodfellow AFB and Keesler AFB was highest following the initial peak at Lackland AFB, and then tapered off. All 3 sites placed ill students on quarters, which resulted in the short-term removal of >600 students from training activities. Only 1 person required hospitalization for adenovirus-associated pneumonia, at Sheppard AFB, during this time (0.01/100 trainees for this 23-week time period). As of October 31, 2007, prevention and control efforts were terminated at Goodfellow AFB and Keesler AFB but continued at Sheppard AFB.

## Discussion

Ad14 spread readily to secondary training sites because of the rapid mobility of BMTs following their graduation from basic training. For the most part, the onset of Ad14-related illness occurred first at sites that received the most BMT graduates. Although Lackland AFB made a concerted effort to identify and segregate outgoing febrile BMTs, more than one quarter of the trainees were likely shedding virus or recovering from illness. In addition, many were possibly preclinical and incubating adenovirus as they departed Lackland AFB, with illness developing shortly after arrival at secondary sites.

Although Ad14 was exported continuously to the secondary sites, neither the ARD rates nor the severity of illness at those sites reached the levels seen at Lackland AFB. Control efforts by Lackland AFB that placed febrile BMTs on medical hold and prevented them from leaving the base seemed to affect severity of illness at Lackland AFB and likely reduced the number of ill persons arriving at secondary sites. The lower rates of illness at secondary sites may also have been due to a decreased number of susceptible persons in the secondary training population, berthing differences that resulted in less contact between trainees at secondary sites, and decreased stress levels among trainees. Additionally, decreased illness severity at the secondary training sites may have resulted from the early identification of patients with suspected cases and their placement on quarters, allowing for rest and recovery. Illness trends at Sheppard AFB tracked Lackland AFB ARD activity most closely, possibly because Sheppard AFB received the largest proportion of BMT graduates compared with other secondary training sites and because healthcare personnel at that base more aggressively identified cases and submitted specimens. Keesler AFB and Goodfellow AFB experienced an earlier decline of infection rates due partially to a lower influx of BMTs from Lackland and partially due to lessening participation in enhanced surveillance efforts after the first 3–4 weeks of surveillance. Adenovirus rates mirrored the overall ARD rates, which suggests that adenovirus accounted for most of the ARD cases during the entire period. As of October 31, 2007, ARD rates had subsided somewhat but not sufficiently to cease surveillance and control efforts.

Surveillance findings indicated that spread of Ad14 to active duty members and dependents outside the training population was minimal, although surveillance efforts were not as robust in these populations. Because secondary training students are segregated from the base population at installations, limited contact takes place between trainees and other military members and their dependents. Still, the lack of spread of this readily transmissible pathogen to persons outside the training population was remarkable.

Eliminating the spread from Lackland AFB to secondary training sites was difficult, because any control measures deployed had to operate within the constraints of the recruit training system. Particularly in wartime, military operational requirements do not permit slowing or canceling the training of new military recruits. Even within this limited setting, segregating ill patients and checking students for fever both before leaving Lackland AFB and upon arrival at their next duty station seemed to affect transmission, because ARD rates remained relatively low at secondary training sites and peak ARD activity did not persist.

A new vaccine for Ad4 and Ad7 is currently under development; phase III has been completed (*14*). The degree of cross-protection this vaccine will offer against Ad14 is unknown, although evidence suggests some protection can be expected. Findings from the Lackland AFB outbreak indicated that Ad7 serum neutralizing antibody was protective against Ad14 illness, mitigating the severity of symptoms (*12*). Previous studies have indicated that Ad4 and Ad7 vaccination was also protective against Ad3 (*8*,*15*) and Ad14 (*8*,*15*–*18*). Thus, implementing the Ad4 and Ad7 vaccine may affect AdARD rates in general in future military trainees.

Following the loss of the Ad4 and Ad7 vaccines, the spread of Ad4 from an Army basic training site to a secondary training installation was associated with a large Ad4 outbreak at a secondary site (*19*). In this report we describe the spread of Ad14 to Air Force secondary training sites by recently graduated basic trainees who moved quickly from Lackland AFB to their next assignment. Military planners must focus on how best to control the spread of infectious respiratory diseases in highly mobile military populations that travel between geographically dispersed locations. Additionally, these planners must consider that rigid public health interventions may be unacceptable because of interference with critical operations. In this instance, we found that interventions could not interfere with the flow of programmed training operations for a military at war. Additionally, detailed studies aimed at better describing the transmission of adenoviruses may result in better focused and more effective control measures. Our results show that public health leaders in both the military and civilian communities should be concerned about the geographic spread of respiratory disease agents by highly mobile populations.
